# Supplementary material for: SARS-CoV-2 humoral and cellular immunity following different combinations of vaccination and breakthrough infection
Source: Nat Commun. 2023 Feb 2;14:572. doi: 10.1038/s41467-023-36250-4 (PMC9894521; doi:10.1038/s41467-023-36250-4)
Supplement: Supplementary file 1 — Supplementary information [file 41467_2023_36250_MOESM1_ESM.pdf]

Supplemental table 1

Pseudonym	Sex	Age	1. vaccination (Mo-Yr)	Vaccine	2. vaccination (Mo-Yr)	Vaccine	3. vaccination (Mo-Yr)	Vaccine	PCR + (Mo-Yr)	Variant	Sampling (Mo-Yr)	Symptoms	Spike ELISA	Nucleocapsid ELISA
2xVacc+α_1	female	21-30	Jan-21	Spikevax	Feb-21	Spikevax	/	/	Mar-21	B.1.1.7	Apr-21	yes	positive	negative
2xVacc+α_2	female	21-30	Feb-21	Spikevax	Mar-21	Spikevax	/	/	Mar-21	B1.1.7	Apr-21	none	positive	negative
2xVacc+α_3	female	21-30	Feb-21	Spikevax	Mar-21	Spikevax	/	/	Mar-21	B1.1.7	Apr-21	none	positive	negative
2xVacc+α_4	female	21-30	Feb-21	Spikevax	Mar-21	Spikevax	/	/	Apr-21	B1.1.7	May-21	yes	positive	positive
2xVacc+α_5	male	31-40	Feb-21	Spikevax	Mar-21	Spikevax	/	/	May-21	B1.1.7	May-21	none	positive	negative
2xVacc+α_6	female	51-60	Jan-21	Spikevax	Feb-21	Spikevax	/	/	Apr-21	B1.1.7	May-21	none	positive	positive
2xVacc+α_7	male	61-70	Jan-21	Spikevax	Feb-21	Spikevax	/	/	Apr-21	B1.1.7	May-21	none	positive	positive
2xVacc+δ_1	male	31-40	Feb-21	Spikevax	Mar-21	Spikevax	/	/	Jun-21	B.1.617.2	Jul-21	none	positive	negative
2xVacc+δ_2	female	21-30	Feb-21	Spikevax	Mar-21	Spikevax	/	/	Jun-21	B.1.617.2	Jul-21	none	positive	negative
2xVacc+δ_3	male	31-40	Feb-21	Spikevax	Mar-21	Spikevax	/	/	Jul-21	B.1.617.2	Aug-21	yes	positive	positive
2xVacc+δ_4	male	21-30	Jan-21	Spikevax	Feb-21	Spikevax	/	/	Jul-21	B.1.617.2	Aug-21	yes	positive	positive
2xVacc+δ_5	female	21-30	Feb-21	Spikevax	Mar-21	Spikevax	/	/	Sep-21	B.1.617.2	Oct-21	yes	positive	negative
2xVacc+δ_6	female	51-60	Feb-21	Spikevax	Mar-21	Spikevax	/	/	Sep-21	B.1.617.2	Oct-21	yes	positive	positive
2xVacc+δ_7	female	51-60	Feb-21	Spikevax	Mar-21	Spikevax	/	/	Sep-21	B.1.617.2	Oct-21	yes	positive	positive
2xVacc+δ_8	female	31-40	Feb-21	Spikevax	Mar-21	Spikevax	/	/	Sep-21	B.1.617.2	Oct-21	yes	positive	positive
2xVacc+δ_9	male	21-30	Feb-21	Spikevax	Mar-21	Spikevax	/	/	Sep-21	B.1.617.2	Oct-21	yes	positive	positive
2xVacc+δ_10	female	31-40	Jan-21	Spikevax	Feb-21	Spikevax	/	/	Sep-21	B.1.617.2	Oct-21	none	positive	positive
2xVacc+δ_11	male	21-30	Apr-21	Spikevax	Jun-21	Spikevax	/	/	Oct-21	B.1.617.2	Nov-21	yes	positive	positive
2xVacc+δ_12	male	21-30	Feb-21	Spikevax	Mar-21	Spikevax	/	/	Oct-21	B.1.617.2	Nov-21	yes	positive	positive
2xVacc+δ_13	female	31-40	Feb-21	Spikevax	Mar-21	Spikevax	/	/	Oct-21	B.1.617.2	Nov-21	yes	positive	positive
3xVacc+o_1	female	31-40	Feb-21	Spikevax	Mar-21	Spikevax	Nov-21	Spikevax	Jan-22	B. 1.1.529	Feb-22	yes	positive	positive
3xVacc+o_2	male	31-40	Jan-21	Spikevax	Mar-21	Spikevax	Nov-21	Spikevax	Jan-22	B. 1.1.529	Feb-22	yes	positive	positive
3xVacc+o_3	male	31-40	Feb-21	Spikevax	Mar-21	Spikevax	Nov-21	Spikevax	Dec-21	B. 1.1.529	Jan-22	yes	positive	positive
3xVacc+o_4	male	31-40	Jan-21	Spikevax	Feb-21	Spikevax	Nov-21	Spikevax	Feb-22	B. 1.1.529	Feb-22	yes	positive	positive
3xVacc+o_5	male	31-40	Jan-21	Spikevax	Feb-21	Spikevax	Nov-21	Spikevax	Jan-22	B. 1.1.529	Mar-22	yes	positive	positive

3xVacc+o_6	female	31-40	Jan-21	Spikevax	Feb-21	Spikevax	Nov-21	Spikevax	Jan-22	B. 1.1.529	Mar-22	yes	positive	positive
3xVacc+o_7	female	31-40	Jan-21	Spikevax	Feb-21	Spikevax	Nov-21	Spikevax	Jan-22	B. 1.1.529	Mar-22	yes	positive	positive
3xVacc+o_8	female	51-60	Feb-21	Spikevax	Mar-21	Spikevax	Oct-21	Spikevax	Feb-22	B. 1.1.529	Mar-22	yes	positive	positive
3xVacc+o_9	male	41-50	Feb-21	Spikevax	Mar-21	Spikevax	Nov-21	Spikevax	Jan-22	B. 1.1.529	Feb-22	yes	positive	positive
3xVacc+o_10	female	51-60	Feb-21	Spikevax	Mar-21	Spikevax	Nov-21	Spikevax	Feb-22	B. 1.1.529	Feb-22	yes	positive	positive
3xVacc+α_1	female	21-30	Jan-21	Spikevax	Feb-21	Spikevax	Dec-21	Comirnaty	Mar-21	B.1.1.7	Jan-22	yes	positive	negative
3xVacc+α_2	female	21-30	Feb-21	Spikevax	Mar-21	Spikevax	Nov-21	Comirnaty	Mar-21	B1.1.7	Dec-21	none	positive	negative
3xVacc+α_3	female	21-30	Feb-21	Spikevax	Mar-21	Spikevax	Dec-21	Comirnaty	Mar-21	B1.1.7	Jan-22	none	positive	negative
3xVacc+α_4	female	21-30	Feb-21	Spikevax	Mar-21	Spikevax	Feb-22	Spikevax	Apr-21	B1.1.7	Mar-22	yes	positive	positive
3xVacc+α_5	male	31-40	Feb-21	Spikevax	Mar-21	Spikevax	Dec-21	Spikevax	May-21	B1.1.7	Jan-22	none	positive	negative
3xVacc+α_6	female	51-60	Jan-21	Spikevax	Feb-21	Spikevax	Nov-21	Spikevax	Apr-21	B1.1.7	Dec-21	none	positive	positive
3xVacc+α_7	male	61-70	Jan-21	Spikevax	Feb-21	Spikevax	Dec-21	Spikevax	Apr-21	B1.1.7	Dec-21	none	positive	positive
3xVacc+δ_1	female	21-30	Feb-21	Spikevax	Mar-21	Spikevax	Nov-21	Spikevax	Jun-21	B.1.617.2	Nov-21	none	positive	negative
3xVacc+δ_2	male	31-40	Feb-21	Spikevax	Mar-21	Spikevax	Nov-21	Spikevax	Jun-21	B.1.617.2	Nov-21	none	positive	negative
3xVacc+δ_3	male	31-40	Feb-21	Spikevax	Mar-21	Spikevax	Nov-21	Spikevax	Jul-21	B.1.617.2	Dec-21	yes	positive	positive
3xVacc+δ_4	female	51-60	Feb-21	Spikevax	Mar-21	Spikevax	Jan-22	Spikevax	Sep-21	B.1.617.2	Mar-22	yes	positive	positive
3xVacc+δ_5	female	51-60	Feb-21	Spikevax	Mar-21	Spikevax	Jan-22	Spikevax	Sep-21	B.1.617.2	Mar-22	yes	positive	positive
3xVacc+δ_6	male	21-30	Apr-21	Spikevax	Jun-21	Spikevax	Jan-22	Spikevax	Oct-21	B.1.617.2	Feb-22	yes	positive	positive
3xVacc+δ_7	female	31-40	Feb-21	Spikevax	Mar-21	Spikevax	Jan-22	Comirnaty	Oct-21	B.1.617.2	Feb-22	yes	positive	positive
3xVacc_1	male	51-60	Feb-21	Spikevax	Mar-21	Spikevax	Oct-21	Spikevax	/	/	Nov-21	/	positive	negative
3xVacc_2	female	31-40	Feb-21	Spikevax	Mar-21	Spikevax	Oct-21	Spikevax	/	/	Nov-21	/	positive	negative
3xVacc_3	male	41-50	Feb-21	Spikevax	Mar-21	Spikevax	Oct-21	Spikevax	/	/	Nov-21	/	positive	negative
3xVacc_4	female	21-30	Feb-21	Spikevax	Mar-21	Spikevax	Oct-21	Spikevax	/	/	Nov-21	/	positive	negative
3xVacc_5	male	51-60	Jan-21	Spikevax	Feb-21	Spikevax	Oct-21	Spikevax	/	/	Nov-21	/	positive	negative
3xVacc_6	female	41-50	Jan-21	Spikevax	Feb-21	Spikevax	Oct-21	Spikevax	/	/	Nov-21	/	positive	negative
3xVacc_7	female	31-40	Feb-21	Spikevax	Mar-21	Spikevax	Oct-21	Spikevax	/	/	Nov-21	/	positive	negative
3xVacc_8	male	41-50	Jan-21	Spikevax	Feb-21	Spikevax	Oct-21	Spikevax	/	/	Nov-21	/	positive	negative
3xVacc_9	female	51-60	Jan-21	Spikevax	Feb-21	Spikevax	Nov-21	Spikevax	/	/	Dec-21	/	positive	negative

3xVacc_10	female	21-30	Feb-21	Spikevax	Mar-21	Spikevax	Nov-21	Comirnaty	/	/	Dec-21	/	positive	negative
3xVacc_11	male	41-50	Jan-21	Spikevax	Feb-21	Spikevax	Nov-21	Spikevax	/	/	Dec-21	/	positive	negative
3xVacc_12	female	51-60	Jan-21	Spikevax	Feb-21	Spikevax	Nov-21	Spikevax	/	/	Dec-21	/	positive	negative
3xVacc_13	male	41-50	Jan-21	Spikevax	Feb-21	Spikevax	Nov-21	Spikevax	/	/	Dec-21	/	positive	negative
3xVacc_14	female	51-60	Feb-21	Spikevax	Mar-21	Spikevax	Nov-21	Spikevax	/	/	Dec-21	/	positive	negative
3xVacc_15	female	51-60	Jan-21	Spikevax	Feb-21	Spikevax	Nov-21	Spikevax	/	/	Dec-21	/	positive	negative
3xVacc_16	male	61-70	Jan-21	Spikevax	Feb-21	Spikevax	Nov-21	Spikevax	/	/	Dec-21	/	positive	negative
3xVacc_17	female	41-50	Feb-21	Spikevax	Mar-21	Spikevax	Nov-21	Spikevax	/	/	Dec-21	/	positive	negative
3xVacc_18	female	61-70	Feb-21	Spikevax	Mar-21	Spikevax	Nov-21	Spikevax	/	/	Dec-21	/	positive	negative
3xVacc_19	female	61-70	Jan-21	Spikevax	Feb-21	Spikevax	Nov-21	Spikevax	/	/	Dec-21	/	positive	negative
3xVacc_20	female	21-30	Mar-21	Spikevax	Apr-21	Spikevax	Nov-21	Spikevax	/	/	Dec-21	/	positive	negative
3xVacc_21	female	31-40	Feb-21	Spikevax	Mar-21	Spikevax	Oct-21	Spikevax	/	/	Dec-21	/	positive	negative
3xVacc_22	female	11-20	Mar-21	Spikevax	Apr-21	Spikevax	Nov-21	Spikevax	/	/	Dec-21	/	positive	negative
3xVacc_23	male	61-70	Jan-21	Spikevax	Feb-21	Spikevax	Nov-21	Spikevax	/	/	Dec-21	/	positive	negative
2xVacc_1	male	41-50	Jan-21	Spikevax	Feb-21	Spikevax	/	/	/	/	Mar-21	/	positive	negative
2xVacc_2	female	61-70	Jan-21	Spikevax	Feb-21	Spikevax	/	/	/	/	Mar-21	/	positive	negative
2xVacc_3	male	61-70	Jan-21	Spikevax	Feb-21	Spikevax	/	/	/	/	Mar-21	/	positive	negative
2xVacc_4	female	41-50	Feb-21	Spikevax	Mar-21	Spikevax	/	/	/	/	Mar-21	/	positive	negative
2xVacc_5	male	41-50	Feb-21	Spikevax	Mar-21	Spikevax	/	/	/	/	Mar-21	/	positive	negative
2xVacc_6	female	51-60	Feb-21	Spikevax	Mar-21	Spikevax	/	/	/	/	Mar-21	/	positive	negative
2xVacc_7	female	31-40	Jan-21	Spikevax	Feb-21	Spikevax	/	/	/	/	Mar-21	/	positive	negative
2xVacc_8	male	31-40	Jan-21	Spikevax	Feb-21	Spikevax	/	/	/	/	Mar-21	/	positive	negative
2xVacc_9	male	41-50	Jan-21	Spikevax	Feb-21	Spikevax	/	/	/	/	Mar-21	/	positive	negative
2xVacc_10	male	31-40	Jan-21	Spikevax	Feb-21	Spikevax	/	/	/	/	Mar-21	/	positive	negative
2xVacc_11	male	61-70	Jan-21	Spikevax	Feb-21	Spikevax	/	/	/	/	Mar-21	/	positive	negative
2xVacc_12	male	41-50	Jan-21	Spikevax	Feb-21	Spikevax	/	/	/	/	Mar-21	/	positive	negative
2xVacc_13	female	51-60	Jan-21	Spikevax	Feb-21	Spikevax	/	/	/	/	Mar-21	/	positive	negative
2xVacc_14	female	41-50	Jan-21	Spikevax	Feb-21	Spikevax	/	/	/	/	Mar-21	/	positive	negative

2xVacc_15	female	21-30	Feb-21	Spikevax	Mar-21	Spikevax	/	/	/	/	Mar-21	/	positive	negative
2xVacc_16	male	41-50	Jan-21	Spikevax	Feb-21	Spikevax	/	/	/	/	Mar-21	/	positive	negative
2xVacc_17	male	41-50	Jan-21	Spikevax	Feb-21	Spikevax	/	/	/	/	Mar-21	/	positive	negative
2xVacc_18	female	61-70	Jan-21	Spikevax	Feb-21	Spikevax	/	/	/	/	Mar-21	/	positive	negative
2xVacc_19	female	31-40	Feb-21	Spikevax	Mar-21	Spikevax	/	/	/	/	Mar-21	/	positive	negative
2xVacc_20	female	51-60	Jan-21	Spikevax	Feb-21	Spikevax	/	/	/	/	Mar-21	/	positive	negative
2xVacc_21	female	31-40	Jan-21	Spikevax	Feb-21	Spikevax	/	/	/	/	Mar-21	/	positive	negative
2xVacc_22	female	21-30	Feb-21	Spikevax	Mar-21	Spikevax	/	/	/	/	Mar-21	/	positive	negative
2xVacc_23	male	61-70	Feb-21	Spikevax	Mar-21	Spikevax	/	/	/	/	Mar-21	/	positive	negative
2xVacc_24	female	31-40	Feb-21	Spikevax	Mar-21	Spikevax	/	/	/	/	Mar-21	/	positive	negative
2xVacc_25	female	21-30	Feb-21	Spikevax	Mar-21	Spikevax	/	/	/	/	Mar-21	/	positive	negative
2xVacc_26	male	51-60	Feb-21	Spikevax	Mar-21	Spikevax	/	/	/	/	Mar-21	/	positive	negative
2xVacc_27	female	41-50	Jan-21	Spikevax	Feb-21	Spikevax	/	/	/	/	Mar-21	/	positive	negative
2xVacc_28	female	21-30	Feb-21	Spikevax	Mar-21	Spikevax	/	/	/	/	Mar-21	/	positive	negative
2xVacc_29	female	31-40	Jan-21	Spikevax	Feb-21	Spikevax	/	/	/	/	Mar-21	/	positive	negative
2xVacc_30	female	51-60	Feb-21	Spikevax	Mar-21	Spikevax	/	/	/	/	Mar-21	/	positive	negative
2xVacc_31	female	51-60	Jan-21	Spikevax	Feb-21	Spikevax	/	/	/	/	Mar-21	/	positive	negative
2xVacc_32	female	51-60	Feb-21	Spikevax	Mar-21	Spikevax	/	/	/	/	Mar-21	/	positive	negative
2xVacc_33	male	31-40	Feb-21	Spikevax	Mar-21	Spikevax	/	/	/	/	Mar-21	/	positive	negative
2xVacc_34	female	41-50	Feb-21	Spikevax	Mar-21	Spikevax	/	/	/	/	Mar-21	/	positive	negative
2xVacc_35	male	21-30	Feb-21	Spikevax	Mar-21	Spikevax	/	/	/	/	Mar-21	/	positive	negative
2xVacc_36	female	41-50	Feb-21	Spikevax	Mar-21	Spikevax	/	/	/	/	Mar-21	/	positive	negative
2xVacc_37	male	41-50	Feb-21	Spikevax	Mar-21	Spikevax	/	/	/	/	Mar-21	/	positive	negative
2xVacc_38	male	21-30	Feb-21	Spikevax	Mar-21	Spikevax	/	/	/	/	Mar-21	/	positive	negative
2xVacc_39	female	21-30	Jan-21	Spikevax	Feb-21	Spikevax	/	/	/	/	Mar-21	/	positive	negative
2xVacc_40	male	41-50	Jan-21	Spikevax	Feb-21	Spikevax	/	/	/	/	Mar-21	/	positive	negative
2xVacc_41	female	41-50	Jan-21	Spikevax	Feb-21	Spikevax	/	/	/	/	Mar-21	/	positive	negative
2xVacc_42	female	51-60	Feb-21	Spikevax	Mar-21	Spikevax	/	/	/	/	Mar-21	/	positive	negative

2xVacc_43	female	51-60	Jan-21	Spikevax	Feb-21	Spikevax	/	/	/	/	Mar-21	/	positive	negative
2xVacc_44	female	51-60	Feb-21	Spikevax	Mar-21	Spikevax	/	/	/	/	Apr-21	/	positive	negative
2xVacc_45	male	21-30	Feb-21	Spikevax	Mar-21	Spikevax	/	/	/	/	Mar-21	/	positive	negative
2xVacc_46	female	61-70	Feb-21	Spikevax	Mar-21	Spikevax	/	/	/	/	Mar-21	/	positive	negative
2xVacc_47	female	61-70	Feb-21	Spikevax	Mar-21	Spikevax	/	/	/	/	Mar-21	/	positive	negative
2xVacc_48	male	31-40	Jan-21	Spikevax	Feb-21	Spikevax	/	/	/	/	Apr-21	/	positive	negative
2xVacc_49	female	41-50	Feb-21	Spikevax	Mar-21	Spikevax	/	/	/	/	Apr-21	/	positive	negative
2xVacc_50	male	21-30	Feb-21	Spikevax	Mar-21	Spikevax	/	/	/	/	Apr-21	/	positive	negative
2xVacc_51	female	51-60	Feb-21	Spikevax	Mar-21	Spikevax	/	/	/	/	Apr-21	/	positive	negative
2xVacc_52	male	41-50	Jan-21	Spikevax	Feb-21	Spikevax	/	/	/	/	Apr-21	/	positive	negative
2xVacc_53	female	61-70	Feb-21	Spikevax	Mar-21	Spikevax	/	/	/	/	Apr-21	/	positive	negative
2xVacc_54	female	41-50	Mar-21	Spikevax	Apr-21	Spikevax	/	/	/	/	May-21	/	positive	negative

Supplemental table 1) Demographics with antigen contact and sampling time points. The table contains following information for each study participant (columns left to right in sequential order): pseudonym, sex, age (as 10-year range), first dose vaccination date, first dose vaccine type, second dose vaccination date, second dose vaccine type, third dose vaccination date, third dose vaccine type, date of positive SARS-CoV-2 RT-PCR, date of sample collection, variant of infection, seropositivity for anti-spike antibodies, seropositivity for anti-nucleocapsid antibodies. Sign “/” indicates that the information is not relevant for the individual.

Supplemental figure 1

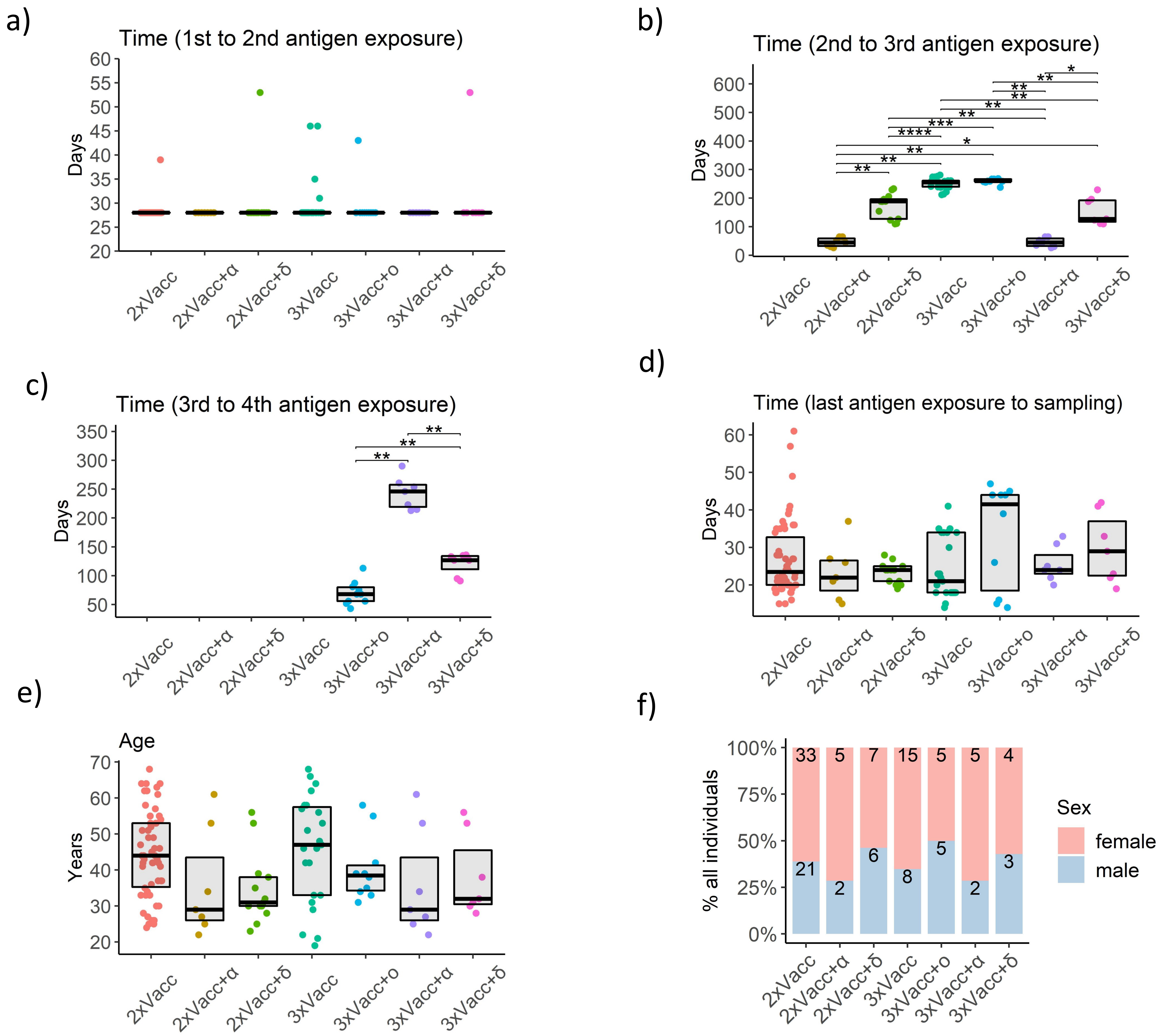

Supplemental Figure 1) Demographic characteristics, the time between sampling, and antigen exposures for the study cohort. a) Time between a) first and second b) second and the third (exact p values in sequential order from the upmost bracket: *p=0.011, **p=0.006, **p=0.006, **p=0.002, **p=0.001, **p=0.004, ***p=0.00088, ****p<0.0001, *p=0.011, **p=0.006, **p=0.001, **p=0.004) c) third and fourth antigen exposure for different antigen exposure groups (exact p values in sequential order from the upmost bracket: **p=0.003, **p=0.003, **p=0.002). d) Time between the last antigen exposure and sampling for different antigen exposure groups. e) Age distribution of the study participants belonging to different antigen exposure groups. Data is displayed as boxplots, indicating the first quartile, median, and third quartile, with individual data points. f) Stacked bar plot representing the sex constitution of the antigen exposure groups. Overlaid numbers indicate the number of individuals of a particular sex for each group. The following numbers of biologically independent samples were included in each group for all the graphs in this figure: 2xVacc, n=54; 2xVacc+α, n=7; 2xVacc+δ, n=13; 3xVacc, n=23; 3xVacc+o, n=10; 3xVacc+α, n=7; 3xVacc+δ, n=7. Differences between the groups were assessed using the two-sided Mann-Whitney test with Holm's correction for multiple testing. Source data are provided as a Source Data file.

Supplemental figure 2

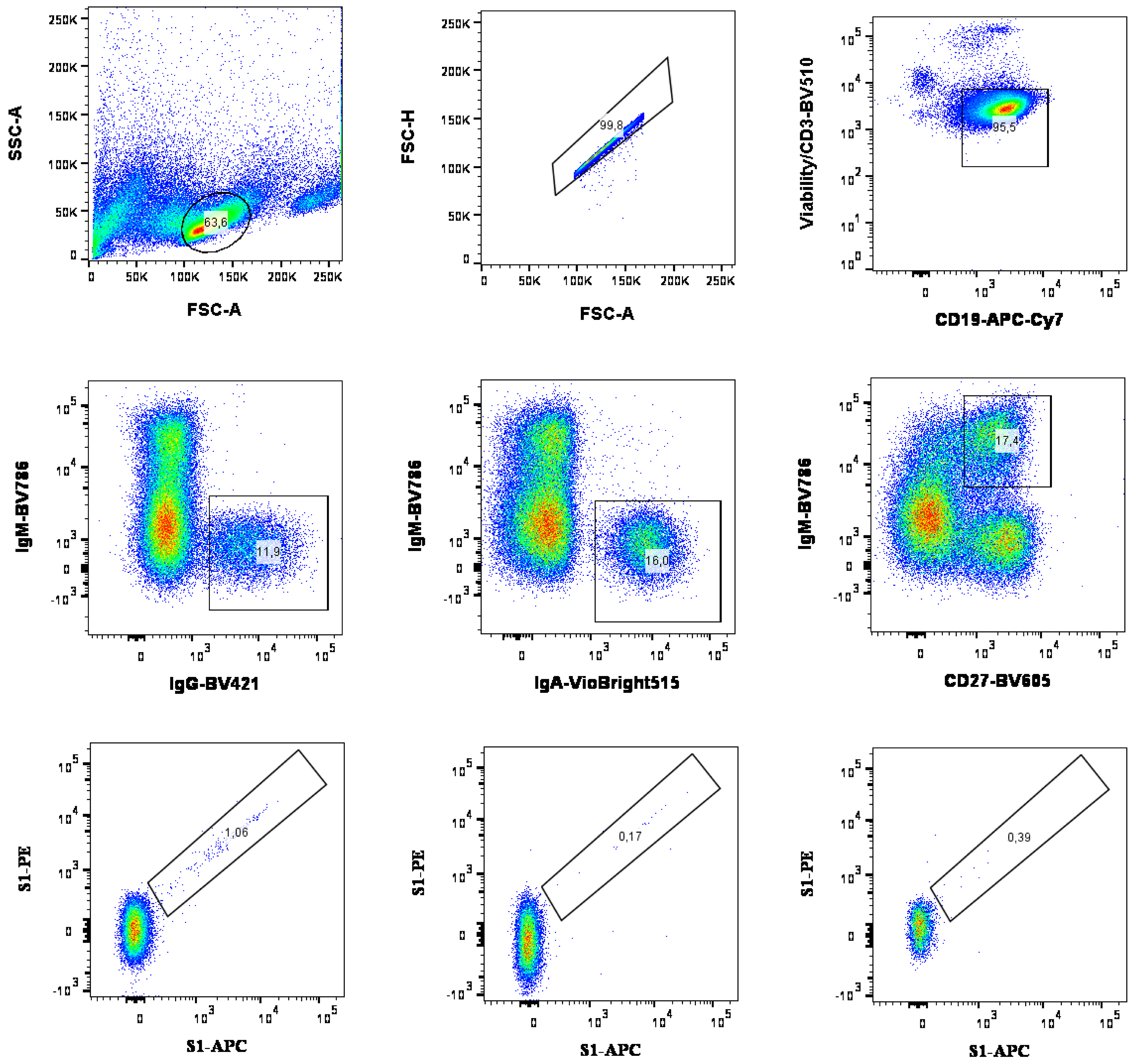

Supplemental Figure 2) Shown is the gating strategy for the identification of S-specific memory B cells. Plots are shown as pseudocolors. Arrows indicate the sequential order in which gates were applied. Numbers within the gates are percentages of the parent population. The above gating strategy was used to derive data presented in figures 3 and 5.

Supplemental figure 3

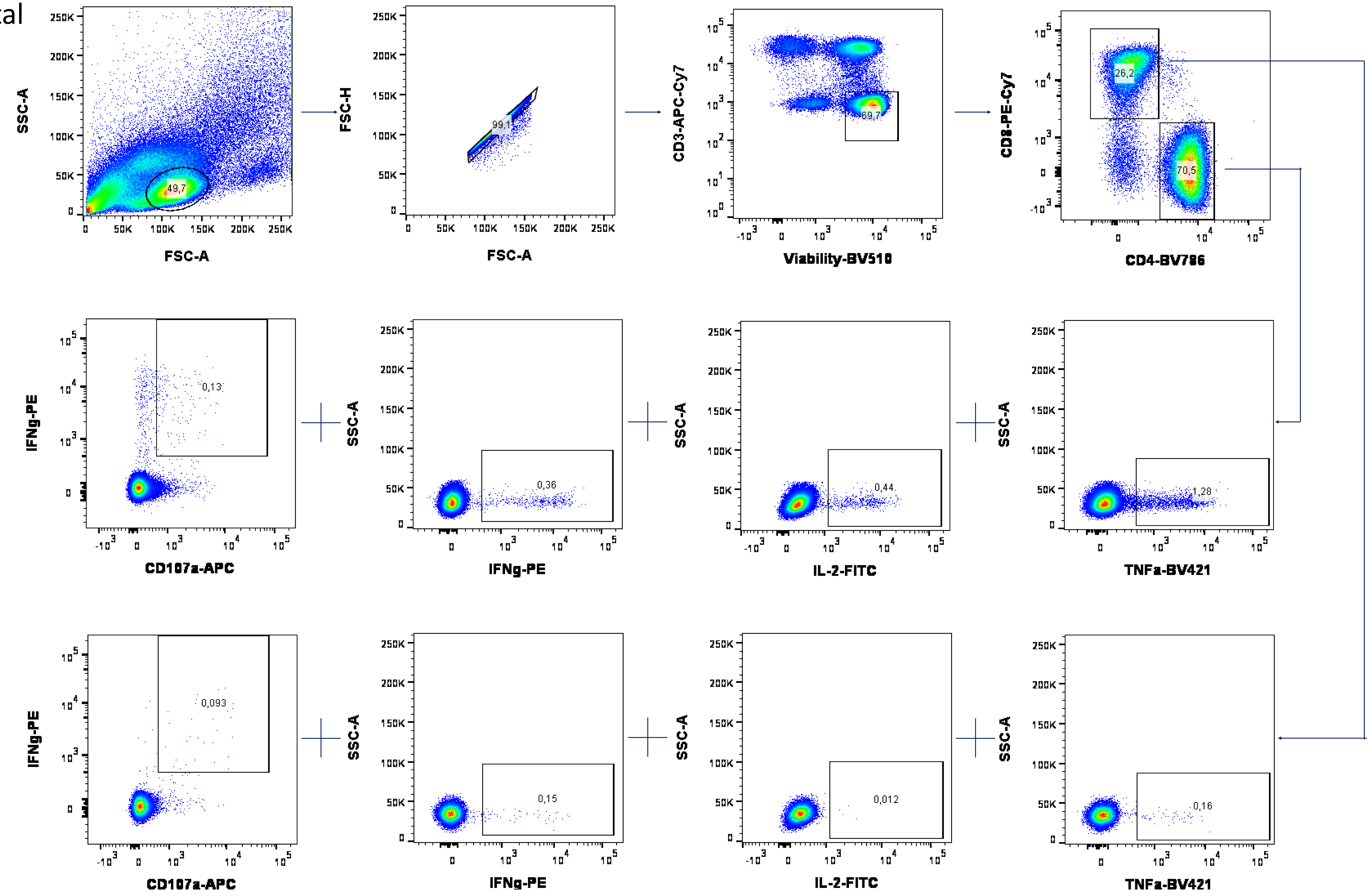

Supplemental Figure 3) Shown is the gating strategy for the identification of antigen-specific T cells. Plots are shown as pseudocolors. Arrows indicate the sequential order in which gates were applied, and the symbol + indicates that the graphs are derived from the same parent population. Numbers within the gates are percentages of the parent population. The above gating strategy was used to derive data presented in figures 4 and 5.
