# Supplementary material for: SARS-CoV-2 humoral and cellular immunity following different combinations of vaccination and breakthrough infection
Source: Nat Commun. 2023 Feb 2;14:572. doi: 10.1038/s41467-023-36250-4 (PMC9894521; doi:10.1038/s41467-023-36250-4)
Supplement: Supplementary file 3 — Description of Additional Supplementary files [file 41467_2023_36250_MOESM3_ESM.docx]

**Supplemental table 1) Demographics with antigen contact and sampling time points.** The table contains following information for each study participant (columns left to right in sequential order): pseudonym, sex, age (as 10-year range), first dose vaccination date, first dose vaccine type, second dose vaccination date, second dose vaccine type, third dose vaccination date, third dose vaccine type, date of positive SARS-CoV-2 RT-PCR, date of sample collection, variant of infection, seropositivity for anti-spike antibodies, seropositivity for anti-nucleocapsid antibodies. Sign “/” indicates that the information is not relevant for the individual.
