# Supplementary material for: SARS-CoV-2 humoral and cellular immunity following different combinations of vaccination and breakthrough infection
Source: Nat Commun. 2023 Feb 2;14:572. doi: 10.1038/s41467-023-36250-4 (PMC9894521; doi:10.1038/s41467-023-36250-4)
Supplement: Supplementary file 4 — Supplementary Data 1 [file 41467_2023_36250_MOESM4_ESM.pdf]

Supplemental table 1

Pseudonym	Sex	Age	1. vaccination (Mo-Yr)	Vaccine	2. vaccination (Mo-Yr)	Vaccine	3. vaccination (Mo-Yr)	Vaccine	PCR + (Mo-Yr)	Variant	Sampling (Mo-Yr)	Symptoms	Spike ELISA	Nucleocapsid ELISA
2xVacc+α_1	female	21-30	Jan-21	Spikevax	Feb-21	Spikevax	/	/	Mar-21	B.1.1.7	Apr-21	yes	positive	negative
2xVacc+α_2	female	21-30	Feb-21	Spikevax	Mar-21	Spikevax	/	/	Mar-21	B1.1.7	Apr-21	none	positive	negative
2xVacc+α_3	female	21-30	Feb-21	Spikevax	Mar-21	Spikevax	/	/	Mar-21	B1.1.7	Apr-21	none	positive	negative
2xVacc+α_4	female	21-30	Feb-21	Spikevax	Mar-21	Spikevax	/	/	Apr-21	B1.1.7	May-21	yes	positive	positive
2xVacc+α_5	male	31-40	Feb-21	Spikevax	Mar-21	Spikevax	/	/	May-21	B1.1.7	May-21	none	positive	negative
2xVacc+α_6	female	51-60	Jan-21	Spikevax	Feb-21	Spikevax	/	/	Apr-21	B1.1.7	May-21	none	positive	positive
2xVacc+α_7	male	61-70	Jan-21	Spikevax	Feb-21	Spikevax	/	/	Apr-21	B1.1.7	May-21	none	positive	positive
2xVacc+δ_1	male	31-40	Feb-21	Spikevax	Mar-21	Spikevax	/	/	Jun-21	B.1.617.2	Jul-21	none	positive	negative
2xVacc+δ_2	female	21-30	Feb-21	Spikevax	Mar-21	Spikevax	/	/	Jun-21	B.1.617.2	Jul-21	none	positive	negative
2xVacc+δ_3	male	31-40	Feb-21	Spikevax	Mar-21	Spikevax	/	/	Jul-21	B.1.617.2	Aug-21	yes	positive	positive
2xVacc+δ_4	male	21-30	Jan-21	Spikevax	Feb-21	Spikevax	/	/	Jul-21	B.1.617.2	Aug-21	yes	positive	positive
2xVacc+δ_5	female	21-30	Feb-21	Spikevax	Mar-21	Spikevax	/	/	Sep-21	B.1.617.2	Oct-21	yes	positive	negative
2xVacc+δ_6	female	51-60	Feb-21	Spikevax	Mar-21	Spikevax	/	/	Sep-21	B.1.617.2	Oct-21	yes	positive	positive
2xVacc+δ_7	female	51-60	Feb-21	Spikevax	Mar-21	Spikevax	/	/	Sep-21	B.1.617.2	Oct-21	yes	positive	positive
2xVacc+δ_8	female	31-40	Feb-21	Spikevax	Mar-21	Spikevax	/	/	Sep-21	B.1.617.2	Oct-21	yes	positive	positive
2xVacc+δ_9	male	21-30	Feb-21	Spikevax	Mar-21	Spikevax	/	/	Sep-21	B.1.617.2	Oct-21	yes	positive	positive
2xVacc+δ_10	female	31-40	Jan-21	Spikevax	Feb-21	Spikevax	/	/	Sep-21	B.1.617.2	Oct-21	none	positive	positive
2xVacc+δ_11	male	21-30	Apr-21	Spikevax	Jun-21	Spikevax	/	/	Oct-21	B.1.617.2	Nov-21	yes	positive	positive
2xVacc+δ_12	male	21-30	Feb-21	Spikevax	Mar-21	Spikevax	/	/	Oct-21	B.1.617.2	Nov-21	yes	positive	positive
2xVacc+δ_13	female	31-40	Feb-21	Spikevax	Mar-21	Spikevax	/	/	Oct-21	B.1.617.2	Nov-21	yes	positive	positive
3xVacc+o_1	female	31-40	Feb-21	Spikevax	Mar-21	Spikevax	Nov-21	Spikevax	Jan-22	B. 1.1.529	Feb-22	yes	positive	positive
3xVacc+o_2	male	31-40	Jan-21	Spikevax	Mar-21	Spikevax	Nov-21	Spikevax	Jan-22	B. 1.1.529	Feb-22	yes	positive	positive
3xVacc+o_3	male	31-40	Feb-21	Spikevax	Mar-21	Spikevax	Nov-21	Spikevax	Dec-21	B. 1.1.529	Jan-22	yes	positive	positive
3xVacc+o_4	male	31-40	Jan-21	Spikevax	Feb-21	Spikevax	Nov-21	Spikevax	Feb-22	B. 1.1.529	Feb-22	yes	positive	positive
3xVacc+o_5	male	31-40	Jan-21	Spikevax	Feb-21	Spikevax	Nov-21	Spikevax	Jan-22	B. 1.1.529	Mar-22	yes	positive	positive

3xVacc+o_6	female	31-40	Jan-21	Spikevax	Feb-21	Spikevax	Nov-21	Spikevax	Jan-22	B. 1.1.529	Mar-22	yes	positive	positive
3xVacc+o_7	female	31-40	Jan-21	Spikevax	Feb-21	Spikevax	Nov-21	Spikevax	Jan-22	B. 1.1.529	Mar-22	yes	positive	positive
3xVacc+o_8	female	51-60	Feb-21	Spikevax	Mar-21	Spikevax	Oct-21	Spikevax	Feb-22	B. 1.1.529	Mar-22	yes	positive	positive
3xVacc+o_9	male	41-50	Feb-21	Spikevax	Mar-21	Spikevax	Nov-21	Spikevax	Jan-22	B. 1.1.529	Feb-22	yes	positive	positive
3xVacc+o_10	female	51-60	Feb-21	Spikevax	Mar-21	Spikevax	Nov-21	Spikevax	Feb-22	B. 1.1.529	Feb-22	yes	positive	positive
3xVacc+α_1	female	21-30	Jan-21	Spikevax	Feb-21	Spikevax	Dec-21	Comirnaty	Mar-21	B.1.1.7	Jan-22	yes	positive	negative
3xVacc+α_2	female	21-30	Feb-21	Spikevax	Mar-21	Spikevax	Nov-21	Comirnaty	Mar-21	B1.1.7	Dec-21	none	positive	negative
3xVacc+α_3	female	21-30	Feb-21	Spikevax	Mar-21	Spikevax	Dec-21	Comirnaty	Mar-21	B1.1.7	Jan-22	none	positive	negative
3xVacc+α_4	female	21-30	Feb-21	Spikevax	Mar-21	Spikevax	Feb-22	Spikevax	Apr-21	B1.1.7	Mar-22	yes	positive	positive
3xVacc+α_5	male	31-40	Feb-21	Spikevax	Mar-21	Spikevax	Dec-21	Spikevax	May-21	B1.1.7	Jan-22	none	positive	negative
3xVacc+α_6	female	51-60	Jan-21	Spikevax	Feb-21	Spikevax	Nov-21	Spikevax	Apr-21	B1.1.7	Dec-21	none	positive	positive
3xVacc+α_7	male	61-70	Jan-21	Spikevax	Feb-21	Spikevax	Dec-21	Spikevax	Apr-21	B1.1.7	Dec-21	none	positive	positive
3xVacc+δ_1	female	21-30	Feb-21	Spikevax	Mar-21	Spikevax	Nov-21	Spikevax	Jun-21	B.1.617.2	Nov-21	none	positive	negative
3xVacc+δ_2	male	31-40	Feb-21	Spikevax	Mar-21	Spikevax	Nov-21	Spikevax	Jun-21	B.1.617.2	Nov-21	none	positive	negative
3xVacc+δ_3	male	31-40	Feb-21	Spikevax	Mar-21	Spikevax	Nov-21	Spikevax	Jul-21	B.1.617.2	Dec-21	yes	positive	positive
3xVacc+δ_4	female	51-60	Feb-21	Spikevax	Mar-21	Spikevax	Jan-22	Spikevax	Sep-21	B.1.617.2	Mar-22	yes	positive	positive
3xVacc+δ_5	female	51-60	Feb-21	Spikevax	Mar-21	Spikevax	Jan-22	Spikevax	Sep-21	B.1.617.2	Mar-22	yes	positive	positive
3xVacc+δ_6	male	21-30	Apr-21	Spikevax	Jun-21	Spikevax	Jan-22	Spikevax	Oct-21	B.1.617.2	Feb-22	yes	positive	positive
3xVacc+δ_7	female	31-40	Feb-21	Spikevax	Mar-21	Spikevax	Jan-22	Comirnaty	Oct-21	B.1.617.2	Feb-22	yes	positive	positive
3xVacc_1	male	51-60	Feb-21	Spikevax	Mar-21	Spikevax	Oct-21	Spikevax	/	/	Nov-21	/	positive	negative
3xVacc_2	female	31-40	Feb-21	Spikevax	Mar-21	Spikevax	Oct-21	Spikevax	/	/	Nov-21	/	positive	negative
3xVacc_3	male	41-50	Feb-21	Spikevax	Mar-21	Spikevax	Oct-21	Spikevax	/	/	Nov-21	/	positive	negative
3xVacc_4	female	21-30	Feb-21	Spikevax	Mar-21	Spikevax	Oct-21	Spikevax	/	/	Nov-21	/	positive	negative
3xVacc_5	male	51-60	Jan-21	Spikevax	Feb-21	Spikevax	Oct-21	Spikevax	/	/	Nov-21	/	positive	negative
3xVacc_6	female	41-50	Jan-21	Spikevax	Feb-21	Spikevax	Oct-21	Spikevax	/	/	Nov-21	/	positive	negative
3xVacc_7	female	31-40	Feb-21	Spikevax	Mar-21	Spikevax	Oct-21	Spikevax	/	/	Nov-21	/	positive	negative
3xVacc_8	male	41-50	Jan-21	Spikevax	Feb-21	Spikevax	Oct-21	Spikevax	/	/	Nov-21	/	positive	negative
3xVacc_9	female	51-60	Jan-21	Spikevax	Feb-21	Spikevax	Nov-21	Spikevax	/	/	Dec-21	/	positive	negative

3xVacc_10	female	21-30	Feb-21	Spikevax	Mar-21	Spikevax	Nov-21	Comirnaty	/	/	Dec-21	/	positive	negative
3xVacc_11	male	41-50	Jan-21	Spikevax	Feb-21	Spikevax	Nov-21	Spikevax	/	/	Dec-21	/	positive	negative
3xVacc_12	female	51-60	Jan-21	Spikevax	Feb-21	Spikevax	Nov-21	Spikevax	/	/	Dec-21	/	positive	negative
3xVacc_13	male	41-50	Jan-21	Spikevax	Feb-21	Spikevax	Nov-21	Spikevax	/	/	Dec-21	/	positive	negative
3xVacc_14	female	51-60	Feb-21	Spikevax	Mar-21	Spikevax	Nov-21	Spikevax	/	/	Dec-21	/	positive	negative
3xVacc_15	female	51-60	Jan-21	Spikevax	Feb-21	Spikevax	Nov-21	Spikevax	/	/	Dec-21	/	positive	negative
3xVacc_16	male	61-70	Jan-21	Spikevax	Feb-21	Spikevax	Nov-21	Spikevax	/	/	Dec-21	/	positive	negative
3xVacc_17	female	41-50	Feb-21	Spikevax	Mar-21	Spikevax	Nov-21	Spikevax	/	/	Dec-21	/	positive	negative
3xVacc_18	female	61-70	Feb-21	Spikevax	Mar-21	Spikevax	Nov-21	Spikevax	/	/	Dec-21	/	positive	negative
3xVacc_19	female	61-70	Jan-21	Spikevax	Feb-21	Spikevax	Nov-21	Spikevax	/	/	Dec-21	/	positive	negative
3xVacc_20	female	21-30	Mar-21	Spikevax	Apr-21	Spikevax	Nov-21	Spikevax	/	/	Dec-21	/	positive	negative
3xVacc_21	female	31-40	Feb-21	Spikevax	Mar-21	Spikevax	Oct-21	Spikevax	/	/	Dec-21	/	positive	negative
3xVacc_22	female	11-20	Mar-21	Spikevax	Apr-21	Spikevax	Nov-21	Spikevax	/	/	Dec-21	/	positive	negative
3xVacc_23	male	61-70	Jan-21	Spikevax	Feb-21	Spikevax	Nov-21	Spikevax	/	/	Dec-21	/	positive	negative
2xVacc_1	male	41-50	Jan-21	Spikevax	Feb-21	Spikevax	/	/	/	/	Mar-21	/	positive	negative
2xVacc_2	female	61-70	Jan-21	Spikevax	Feb-21	Spikevax	/	/	/	/	Mar-21	/	positive	negative
2xVacc_3	male	61-70	Jan-21	Spikevax	Feb-21	Spikevax	/	/	/	/	Mar-21	/	positive	negative
2xVacc_4	female	41-50	Feb-21	Spikevax	Mar-21	Spikevax	/	/	/	/	Mar-21	/	positive	negative
2xVacc_5	male	41-50	Feb-21	Spikevax	Mar-21	Spikevax	/	/	/	/	Mar-21	/	positive	negative
2xVacc_6	female	51-60	Feb-21	Spikevax	Mar-21	Spikevax	/	/	/	/	Mar-21	/	positive	negative
2xVacc_7	female	31-40	Jan-21	Spikevax	Feb-21	Spikevax	/	/	/	/	Mar-21	/	positive	negative
2xVacc_8	male	31-40	Jan-21	Spikevax	Feb-21	Spikevax	/	/	/	/	Mar-21	/	positive	negative
2xVacc_9	male	41-50	Jan-21	Spikevax	Feb-21	Spikevax	/	/	/	/	Mar-21	/	positive	negative
2xVacc_10	male	31-40	Jan-21	Spikevax	Feb-21	Spikevax	/	/	/	/	Mar-21	/	positive	negative
2xVacc_11	male	61-70	Jan-21	Spikevax	Feb-21	Spikevax	/	/	/	/	Mar-21	/	positive	negative
2xVacc_12	male	41-50	Jan-21	Spikevax	Feb-21	Spikevax	/	/	/	/	Mar-21	/	positive	negative
2xVacc_13	female	51-60	Jan-21	Spikevax	Feb-21	Spikevax	/	/	/	/	Mar-21	/	positive	negative
2xVacc_14	female	41-50	Jan-21	Spikevax	Feb-21	Spikevax	/	/	/	/	Mar-21	/	positive	negative

2xVacc_15	female	21-30	Feb-21	Spikevax	Mar-21	Spikevax	/	/	/	/	Mar-21	/	positive	negative
2xVacc_16	male	41-50	Jan-21	Spikevax	Feb-21	Spikevax	/	/	/	/	Mar-21	/	positive	negative
2xVacc_17	male	41-50	Jan-21	Spikevax	Feb-21	Spikevax	/	/	/	/	Mar-21	/	positive	negative
2xVacc_18	female	61-70	Jan-21	Spikevax	Feb-21	Spikevax	/	/	/	/	Mar-21	/	positive	negative
2xVacc_19	female	31-40	Feb-21	Spikevax	Mar-21	Spikevax	/	/	/	/	Mar-21	/	positive	negative
2xVacc_20	female	51-60	Jan-21	Spikevax	Feb-21	Spikevax	/	/	/	/	Mar-21	/	positive	negative
2xVacc_21	female	31-40	Jan-21	Spikevax	Feb-21	Spikevax	/	/	/	/	Mar-21	/	positive	negative
2xVacc_22	female	21-30	Feb-21	Spikevax	Mar-21	Spikevax	/	/	/	/	Mar-21	/	positive	negative
2xVacc_23	male	61-70	Feb-21	Spikevax	Mar-21	Spikevax	/	/	/	/	Mar-21	/	positive	negative
2xVacc_24	female	31-40	Feb-21	Spikevax	Mar-21	Spikevax	/	/	/	/	Mar-21	/	positive	negative
2xVacc_25	female	21-30	Feb-21	Spikevax	Mar-21	Spikevax	/	/	/	/	Mar-21	/	positive	negative
2xVacc_26	male	51-60	Feb-21	Spikevax	Mar-21	Spikevax	/	/	/	/	Mar-21	/	positive	negative
2xVacc_27	female	41-50	Jan-21	Spikevax	Feb-21	Spikevax	/	/	/	/	Mar-21	/	positive	negative
2xVacc_28	female	21-30	Feb-21	Spikevax	Mar-21	Spikevax	/	/	/	/	Mar-21	/	positive	negative
2xVacc_29	female	31-40	Jan-21	Spikevax	Feb-21	Spikevax	/	/	/	/	Mar-21	/	positive	negative
2xVacc_30	female	51-60	Feb-21	Spikevax	Mar-21	Spikevax	/	/	/	/	Mar-21	/	positive	negative
2xVacc_31	female	51-60	Jan-21	Spikevax	Feb-21	Spikevax	/	/	/	/	Mar-21	/	positive	negative
2xVacc_32	female	51-60	Feb-21	Spikevax	Mar-21	Spikevax	/	/	/	/	Mar-21	/	positive	negative
2xVacc_33	male	31-40	Feb-21	Spikevax	Mar-21	Spikevax	/	/	/	/	Mar-21	/	positive	negative
2xVacc_34	female	41-50	Feb-21	Spikevax	Mar-21	Spikevax	/	/	/	/	Mar-21	/	positive	negative
2xVacc_35	male	21-30	Feb-21	Spikevax	Mar-21	Spikevax	/	/	/	/	Mar-21	/	positive	negative
2xVacc_36	female	41-50	Feb-21	Spikevax	Mar-21	Spikevax	/	/	/	/	Mar-21	/	positive	negative
2xVacc_37	male	41-50	Feb-21	Spikevax	Mar-21	Spikevax	/	/	/	/	Mar-21	/	positive	negative
2xVacc_38	male	21-30	Feb-21	Spikevax	Mar-21	Spikevax	/	/	/	/	Mar-21	/	positive	negative
2xVacc_39	female	21-30	Jan-21	Spikevax	Feb-21	Spikevax	/	/	/	/	Mar-21	/	positive	negative
2xVacc_40	male	41-50	Jan-21	Spikevax	Feb-21	Spikevax	/	/	/	/	Mar-21	/	positive	negative
2xVacc_41	female	41-50	Jan-21	Spikevax	Feb-21	Spikevax	/	/	/	/	Mar-21	/	positive	negative
2xVacc_42	female	51-60	Feb-21	Spikevax	Mar-21	Spikevax	/	/	/	/	Mar-21	/	positive	negative

2xVacc_43	female	51-60	Jan-21	Spikevax	Feb-21	Spikevax	/	/	/	/	Mar-21	/	positive	negative
2xVacc_44	female	51-60	Feb-21	Spikevax	Mar-21	Spikevax	/	/	/	/	Apr-21	/	positive	negative
2xVacc_45	male	21-30	Feb-21	Spikevax	Mar-21	Spikevax	/	/	/	/	Mar-21	/	positive	negative
2xVacc_46	female	61-70	Feb-21	Spikevax	Mar-21	Spikevax	/	/	/	/	Mar-21	/	positive	negative
2xVacc_47	female	61-70	Feb-21	Spikevax	Mar-21	Spikevax	/	/	/	/	Mar-21	/	positive	negative
2xVacc_48	male	31-40	Jan-21	Spikevax	Feb-21	Spikevax	/	/	/	/	Apr-21	/	positive	negative
2xVacc_49	female	41-50	Feb-21	Spikevax	Mar-21	Spikevax	/	/	/	/	Apr-21	/	positive	negative
2xVacc_50	male	21-30	Feb-21	Spikevax	Mar-21	Spikevax	/	/	/	/	Apr-21	/	positive	negative
2xVacc_51	female	51-60	Feb-21	Spikevax	Mar-21	Spikevax	/	/	/	/	Apr-21	/	positive	negative
2xVacc_52	male	41-50	Jan-21	Spikevax	Feb-21	Spikevax	/	/	/	/	Apr-21	/	positive	negative
2xVacc_53	female	61-70	Feb-21	Spikevax	Mar-21	Spikevax	/	/	/	/	Apr-21	/	positive	negative
2xVacc_54	female	41-50	Mar-21	Spikevax	Apr-21	Spikevax	/	/	/	/	May-21	/	positive	negative
