# Supplementary material for: SARS-CoV-2 humoral and cellular immunity following different combinations of vaccination and breakthrough infection
Source: Nat Commun. 2023 Feb 2;14:572. doi: 10.1038/s41467-023-36250-4 (PMC9894521; doi:10.1038/s41467-023-36250-4)
Supplement: Supplementary file 5 — Reporting Summary [file 41467_2023_36250_MOESM5_ESM.pdf]

nature portfolio

Corresponding author(s): Prof. Dr. Hendrik Streeck

Last updated by author(s): 06.01.2023

Reporting Summary

Nature Portfolio wishes to improve the reproducibility of the work that we publish. This form provides structure for consistency and transparency in reporting. For further information on Nature Portfolio policies, see our Editorial Policies and the Editorial Policy Checklist.

Statistics

For all statistical analyses, confirm that the following items are present in the figure legend, table legend, main text, or Methods section.

n/a	Confirmed	
☐	☒	The exact sample size (*n*) for each experimental group/condition, given as a discrete number and unit of measurement
☐	☒	A statement on whether measurements were taken from distinct samples or whether the same sample was measured repeatedly
☐	☒	The statistical test(s) used AND whether they are one- or two-sided<br>*Only common tests should be described solely by name; describe more complex techniques in the Methods section.*
☐	☒	A description of all covariates tested
☐	☒	A description of any assumptions or corrections, such as tests of normality and adjustment for multiple comparisons
☐	☒	A full description of the statistical parameters including central tendency (e.g. means) or other basic estimates (e.g. regression coefficient) AND variation (e.g. standard deviation) or associated estimates of uncertainty (e.g. confidence intervals)
☐	☒	For null hypothesis testing, the test statistic (e.g. *F*, *t*, *r*) with confidence intervals, effect sizes, degrees of freedom and *P* value noted<br>*Give P values as exact values whenever suitable.*
☒	☐	For Bayesian analysis, information on the choice of priors and Markov chain Monte Carlo settings
☒	☐	For hierarchical and complex designs, identification of the appropriate level for tests and full reporting of outcomes
☐	☒	Estimates of effect sizes (e.g. Cohen's *d*, Pearson's *r*), indicating how they were calculated

Our web collection on statistics for biologists contains articles on many of the points above.

Software and code

Policy information about availability of computer code

Data collection	FACSDiva™ Software Version 8.0 (BD Bioscience)
Data analysis	RStudio 2021.09.0 Build 351, GraphPad Prism software version 9.4.1. (681), FlowJo Software version 10.0.7 (TreeStar)

For manuscripts utilizing custom algorithms or software that are central to the research but not yet described in published literature, software must be made available to editors and reviewers. We strongly encourage code deposition in a community repository (e.g. GitHub). See the Nature Portfolio guidelines for submitting code & software for further information.

Data

Policy information about availability of data

All manuscripts must include a data availability statement. This statement should provide the following information, where applicable:

- Accession codes, unique identifiers, or web links for publicly available datasets
- A description of any restrictions on data availability
- For clinical datasets or third party data, please ensure that the statement adheres to our policy

The data contain information that could compromise the privacy of research participants. Data sharing restrictions imposed by national and transnational data protection laws prohibit the general sharing of data. However, upon submission of a proposal to the corresponding author and approval of this proposal by (i) the principal investigator, (ii) the Ethics Committee of the University of Bonn, and (iii) the data protection officer of the University Hospital Bonn, data collected for the study can be made available to other researchers. A source data file containing the statistics presented in the figures and a Supplemental table containing demographic information are provided with this paper.

Human research participants

Policy information about studies involving human research participants and Sex and Gender in Research.

Reporting on sex and gender	Individuals were not discriminated by sex or gender when recruiting them for the study and allocating them to groups, however, we collected information about sex and gender. Moreover, the study was not designed to perform sex- or gender-based analyses.
Population characteristics	The information we collected regarding population characteristics is available in supplemental table 1
Recruitment	A total of 110 individuals that were initially immunized with 2 doses of mRNA-based SARS-CoV-2 vaccine and subsequently infected and/or vaccinated were recruited for the study. The recruitment was conducted by the occupational healthcare department of the University Hospital Bonn. The first contact was established by telephone after which a written invitation and a consent form was sent to the participants. All individuals were sampled 2-9 weeks following the last antigen exposure. The individuals belonging to different study arms were preselected so that the times from the last antigen exposure did not significantly differ between the groups. Age or sex was not among the selection criteria and no significant differences in age and sex distribution were observed between the groups. Detailed information on the antigen exposure and sampling time points as well as demographic information is provided in Supplemental figure 1 and Supplemental table 1. Breakthrough infections were confirmed by RT-PCR and the viral RNA was sequenced as a part of routine SARS-CoV-2 variant monitoring at the diagnostics department of the Institute of Virology, University Hospital Bonn. All participants were either employed or studied at the University Hospital Bonn at the time of sampling but were not necessarily healthcare workers. As employees of the University Hospital Bonn study participants were obliged to perform two antigen tests every week and RT-PCR whenever they developed symptoms similar to Covid-19. Furthermore, individuals with a history of previous SARS-CoV-2 infection were not taken into the study. All individuals that had a breakthrough infection were infected only once. For the groups without breakthrough infections, only individuals without confirmed SARS-CoV-2 infection, and negative nucleocapsid ELISA results were included.
Ethics oversight	The study was approved by the Ethics Committee of the Medical Faculty of the University of Bonn (ethics approval numbers 125/21) and all participants provided written informed consent. No compensation was provided for the participants.

Note that full information on the approval of the study protocol must also be provided in the manuscript.

Field-specific reporting

Please select the one below that is the best fit for your research. If you are not sure, read the appropriate sections before making your selection.

☒ Life sciences ☐ Behavioural & social sciences ☐ Ecological, evolutionary & environmental sciences

For a reference copy of the document with all sections, see nature.com/documents/nr-reporting-summary-flat.pdf

Life sciences study design

All studies must disclose on these points even when the disclosure is negative.

Sample size	Sample sizes were determined by availability of the samples.
Data exclusions	No data were excluded
Replication	Standards and/or controls were measured during each experiments to ensure reproducibility or the method. ELISA experiments were performed in two replicates. Plasma neutralization capacity was assessed by measuring 10 dilutions of each sample. For flow cytometry experiments, no technical replicates were performed due to the scarcity of the samples.

Randomization	Participants were grouped based on their SARS-CoV-2 immunizations status
Blinding	Investigators received unique identifier codes (no personal names) for the samples, allocation to groups was done during the data analysis

Reporting for specific materials, systems and methods

We require information from authors about some types of materials, experimental systems and methods used in many studies. Here, indicate whether each material, system or method listed is relevant to your study. If you are not sure if a list item applies to your research, read the appropriate section before selecting a response.

Materials & experimental systems

n/a	Involved in the study	
☐	☒	Antibodies
☐	☒	Eukaryotic cell lines
☒	☐	Palaeontology and archaeology
☒	☐	Animals and other organisms
☐	☒	Clinical data
☒	☐	Dual use research of concern

Methods

n/a	Involved in the study	
☒	☐	ChIP-seq
☐	☒	Flow cytometry
☒	☐	MRI-based neuroimaging

Antibodies

Antibodies used	HRP-conjugated anti-IgG antibody (Goat anti-Human IgG (Heavy chain) Secondary Antibody, HRP, Invitrogen, A18805) diluted 1:8000; HRP-conjugated anti-IgA antibody (Goat anti-Human IgA (Heavy chain) Secondary Antibody, HRP, Invitrogen, A18781) diluted 1:1000, anti-SARS-CoV-2-RBD antibody, clone CR3022, Abcam, ab278112/ab273073 (15 ng/ml); BD FastImmune™ CD28/CD49d, BD, 347690, 1 µg/ml); anti-CD107a-APC (clone H4A3; Biolegend, 328620, diluted 1:40); anti-CD3-APC-Cy7 (clone UCHT1; Biolegend, 300426, diluted 1:40), anti-CD4-BV786 (clone SK3; BD Bioscience, 344642, diluted 1:40), anti-IFNγ-PE (clone B27; Biolegend, 506507, diluted 1:40), anti-TNFα-BV421 (clone Mab11; Biolegend, 502932, diluted 1:80), and anti-IL2-AF488 (clone MQ1-17H12; Biolegend, 500304, diluted 1:20); anti-IgG-BV421 antibody (clone G18-145, Biolegend, 562581, diluted 1:20); antibodies blocking human Fc receptors (FcR block, Miltenyi Biotec, 130-059-901, diluted 1:10); anti-CD3-BV510 (clone UCHT1, Biolegend, 300448, diluted 1:40), anti-CD27-BV605 (clone O323, Biolegend, 302830, diluted 1:20), anti-IgM-BV785 (clone MHM-88, Biolegend, 314544, diluted 1:20), anti-IgA-VioBright 515 (clone REA1014, Miltenyi Biotec, 130-116-886, diluted 1:40), anti-CD21-PE-Cy7 (clone Bu32, Biolegend, 354912, diluted 1:160), and anti-CD19-APC-Cy7 (clone HIB19, Biolegend, 302218, diluted 1:80).
Validation	For flow cytometry experiments antibodies were titrated in following manner: For every fluorescently-labeled antibody eight 2-fold dilutions starting from 1:10 were prepared and incubated with PBMC of healthy donors. The sampels were stained 15min at 4°C, washed with PBS and acquired on flow cytometer. To find the optimal concentration staining index was calculated as the ratio of the separation between the positive population and the negative population (difference in MFIs), divided by two times the standard deviation of the negative population. For ELISA experiments secondary antibodies conjugated to HRP were tested as follows: The overall ELISA procedure was performed as described in the manuscript. Seven dilutions of a plasma sample confirmed seropositive for anti-SARS-CoV-2 IgG were prepared and applied to the rows of a pre-coated 96-well ELISA plate. Subsequently, eleven 2-fold dilutions of secondary HRP-conjugated antibodies starting with 1:250 and a negative control without the antibody were added to the plate columns. The ELISA was than developed as described. The highest secondary antibody dilution that gave maximal signal increase over the background (no sample control) for most of the sample dilutions was used in the further assays.

Eukaryotic cell lines

Policy information about cell lines and Sex and Gender in Research

Cell line source(s)	Vero E6; ATCC
Authentication	Cell line was not authenticated
Mycoplasma contamination	Cell line was negative for mycoplasma
Commonly misidentified lines (See ICLAC register)	No commonly misidentified cell line was used for the study.

Clinical data

Policy information about clinical studies
All manuscripts should comply with the ICMJEguidelines for publication of clinical research and a completedCONSORT checklist must be included with all submissions.

Clinical trial registration	Study does not involve clinical trials/clinical trial associated data.
Study protocol	Study does not involve clinical trials/clinical trial associated data.
Data collection	Study does not involve clinical trials/clinical trial associated data.
Outcomes	Study does not involve clinical trials/clinical trial associated data.

Flow Cytometry

Plots

Confirm that:

- [x] The axis labels state the marker and fluorochrome used (e.g. CD4-FITC).
- [x] The axis scales are clearly visible. Include numbers along axes only for bottom left plot of group (a 'group' is an analysis of identical markers).
- [x] All plots are contour plots with outliers or pseudocolor plots.
- [x] A numerical value for number of cells or percentage (with statistics) is provided.

Methodology

Sample preparation	Human PBMCs were used as samples, for processing details please look at the Methods section of the manuscript
Instrument	BD FACS Celesta, IC.nr:94915
Software	FACSDiva™ Software Version 8.0 (BD Bioscience), FlowJo Software version 10.0.7 (TreeStar)
Cell population abundance	No sorting was performed
Gating strategy	Currently there is no detailed gating startegy provided as supplemental material. We can provide gating strategy upon editors/revieweres request

- [x] Tick this box to confirm that a figure exemplifying the gating strategy is provided in the Supplementary Information.
